# Risk profile of postnatal women and their babies attending a rural district hospital in South Africa

**DOI:** 10.3389/fgwh.2022.1024936

**Published:** 2022-12-16

**Authors:** Kate Rees, Chipo Mutyambizi, Rendani Ndou, Helen E Struthers, James A McIntyre, Jackie Dunlop

**Affiliations:** ^1^ Anova Health Institute, Johannesburg, South Africa; ^2^ Department of Community Health, School of Public Health, Faculty of Health Sciences, University of the Witwatersrand, Johannesburg, South Africa; ^3^ Division of Infectious Diseases & HIV Medicine, Department of Medicine, University of Cape Town, Cape Town, South Africa; ^4^ School of Public Health and Family Medicine, Faculty of Health Sciences, University of Cape Town, Cape Town, South Africa; ^5^ Division of Community Paediatrics, Department of Paediatrics and Child Health, School of Clinical Medicine, Faculty of Health Sciences, University of the Witwatersrand, Johannesburg, South Africa

**Keywords:** postnatal, pMTCT, rural health, postnatal mental health, postnatal care

## Abstract

**Background:**

Maternal and neonatal mortality remain unacceptably high and inequitably distributed in South Africa, with the postnatal period being a dangerous time for both mother and baby. The aim of this paper is to describe the risk factors for poor postnatal outcomes, including postnatal mental health disorders, in a population of postnatal women and their babies utilising rural district hospital services in Limpopo Province, with a focus on HIV. We also describe health care provider compliance with relevant guidelines.

**Methods:**

All women discharged from the postnatal ward of the district hospital who consented to participate were enrolled. A research nurse used a structured questionnaire to collect data about sociodemographic information, pregnancy and pre-existing conditions, complications during labour and birth, pregnancy outcomes and mental health risk factors.

**Results:**

The questionnaire was completed for 882 women at the time of discharge. Only 354 (40.2%) of participants had completed secondary education, and 105 (11.9%) reported formal employment. Chronic hypertension was recorded in 20 women (2.3%), with an additional 49 (5.6%) developing a hypertensive disorder during pregnancy. HIV prevalence was 22.8%. 216 women (24.5%) had a mental health risk factor, with 40 reporting more than one (4.5%). Having no income, no antenatal care, having HIV and any hypertensive disorder were significantly associated with a positive mental health risk screen in multivariable analysis. There were 31 stillbirths and early neonatal deaths (3.5%), and 119 babies (13.4%) were born at a low birth weight. Stillbirth or early neonatal death was significantly associated with no antenatal care in multivariable analysis.

**Conclusions:**

Women and babies in this study experienced multiple risk factors for poor outcomes in the postpartum period. Postnatal care should be strengthened in order to address the dominant risks to mothers and babies, including socioeconomic challenges, HIV and hypertension, and risks to mental health. Tools to identify mothers and babies at risk of postnatal complications would allow limited resources to be allocated where they are most needed.

## Introduction

The last decade has seen substantial improvements in maternal and neonatal mortality in South Africa. Since 2009, the maternal mortality ratio (MMR) and neonatal mortality rate have declined, largely due to increased coverage of antiretroviral therapy and prevention of mother to child transmission of HIV (1). However, the country did not meet the Millennium Development Goals, and mortality in these groups remains unacceptably high, and inequitably distributed (2). In general, rural areas bear a higher burden than urban areas.

The Saving Mothers Report (SMR) examines maternal death data provided by South African health facilities, with the most recent report covering the years 2014 to 2016 (3). Over this period, approximately 20% of maternal deaths occurred outside of health facilities. Since an estimated 96% of women give birth in health facilities (4), these deaths occur largely in women who have been discharged after birth (in the postnatal period). The SMR emphasizes that postnatal care has been neglected in South Africa and needs a renewed focus (3). Non-pregnancy related infection remains the leading cause of maternal death in all provinces, in hospital and out of hospital, and 90% of these deaths are in women living with HIV. Obstetric hemorrhage and hypertensive disorders are the next most common causes of death (3).

Neonatal deaths also remain high. In 2015, facility-based reports from the District Health Information System (DHIS) show a rate of 12 per 1,000 live births, but a population-based survey (SADHS) estimated the rate at 21 per 1,000 live births (5). Out of facility deaths may account for some of this discrepancy, but out of facility deaths are not routinely monitored and little is known about them (5). Prematurity is the most common cause of neonatal death, followed by intra-partum related events (such as birth asphyxia). Improving postnatal care, including the provision of quality postnatal care in the community, has the potential to impact positively on child health and survival (5).

Although not a common cause of mortality, mental health is also critically important in the postpartum period and causes substantial morbidity. Depression and anxiety are common in South African women at this time (6), and when unrecognized and untreated can impact very negatively on both mothers' and babies' health. The structural environment in which South African women live contributes to these high rates (7), with poverty, gender-based violence and lack of social support influencing the development of common postnatal mental disorders.

This paper is derived from a study conducted in one sub-district of Mopani District, focused on the postnatal period. The aim of the paper is to describe a population of postnatal women and their babies utilizing district hospital services in a rural district of South Africa. Specifically, we aim to describe their risk factors for poor postnatal outcomes, including postnatal mental health disorders, with a focus on maternal and infant HIV status. We also describe health care provider compliance with the South African Guidelines for the Prevention of Mother to Child Transmission of Communicable Infections.. Understanding the risks facing women and babies in the period immediately following discharge from birth centers could help to design postnatal services that meet their needs and close existing gaps in service delivery.

## Methods

This analysis forms part of a randomized controlled trial (RCT) study. The primary objective of the RCT was to determine whether an additional postnatal contact at 7–14 days (in line with WHO recommendations), to a subset of high-risk mother-infant pairs, leads to improved care and the early detection and management of complications in high-risk mother-infant pairs. This objective is addressed in our previously published paper on the outcomes of the intervention (8). As a secondary objective we now seek to describe the risk factors for poor outcomes amongst this population of mothers.

### Setting

Mopani District is a rural district in Limpopo Province, South Africa, with a population of approximately 1.2 million people (two percent of the South African population). Compared to the rest of South Africa, the population has a higher proportion of children, and lower proportion of adults of working age (9). It falls into the second socioeconomic quintile in South Africa (second poorest quintile) (10). Limpopo Province has a MMR 15% higher than the South African average. There are eight hospitals in the district, including five district hospitals, feeding into one regional hospital. There are no user fees at point of service for pregnant women. Data for the study was collected at Maphutha L Malatiji Hospital (MLMH). MLMH is a district hospital and is the only hospital in Ba-Phalaborwa sub-district. Although births also occur at the one Community Health Centre, the majority of births in the area take place at the district hospital. In 2019, there were 25 333 live births in the district, 71% of which took place at district hospitals (11).

### Sample size

In order to address the larger study objective, the sample size calculation for detecting a difference between two proportions was used. Assuming a sample proportion in the intervention group of 15% and 10% in the control group, a power of 80% and confidence interval of 95%, the sample size was estimated at 683 mothers. To account for the possibility of refusals we added another 10% to this number.

### Data collection

Data collection was conducted between August 2020 and January 2021 at MLMH. All mothers who were discharged from the postnatal ward of the facility during this period were invited to participate. A research nurse based in the postnatal ward of the district hospital explained the study to every woman following birth, at the time of discharge. Mothers received no incentives to participate in the study. All women who consented were recruited into the study. Women were excluded if (1) they chose not to participate (2) were under the age of 18 years and not accompanied by a parent or guardian (3) lived outside Ba-Phalaborwa sub-district. Women under 18 years of age were only enrolled if a parent or guardian gave consent, and the adolescent provided assent.

The research nurse completed the risk assessment tool with each participant. To overcome language barriers and ensure data accuracy, data was collected in the respondent's preferred language. Data quality checks were conducted on a weekly basis. Information was captured from discharge summaries, and when variables were not available in discharge summaries, was self-reported. Any woman being discharged from the postnatal ward of the hospital was eligible to participate, regardless of the outcome of the pregnancy. Data was collected and managed using REDCap data tools (12, 13) hosted at Anova Health Institute.

Nine hundred and six women were assessed for eligibility, 21 were ineligible (six lived outside the study area, and 15 parents/guardians were unavailable to consent) leaving 885 eligible women. Three eligible women declined to participate, resulting in final participation of 882 women or 99.7%.

### Intervention and instruments

The RCT intervention was an additional structured telephonic follow-up, made at between 7 and 14 days after birth. All participants were again followed up at three months following enrollment to collect outcomes data. The RCT made use of the following instruments: a risk assessment tool, 7–14-day check tool, a follow up tool, 3 months follow up tool and a file review. File reviews were done for two reasons: firstly, to investigate the circumstances of perinatal deaths, and secondly to investigate service utilization after discharge. This analysis draws on data collected from the RCT study and makes use of cross-sectional data collected using the risk assessment tool and the file reviews conducted for perinatal deaths. Data was collected using the risk assessment tool at the time of discharge from the postnatal ward (after six hours for a normal vaginal birth or 36–48 h for a caesarian birth*).* This was before the intervention and could not have been impacted by the intervention. This analysis does not include data on three post-discharge infant deaths. There were no post-discharge maternal deaths amongst participants. All other tools are described in our previously published paper on the outcomes of the 7–14-day call (8).

### Risk assessment tool

Mother-infant pairs recruited into the study were assessed for potential risk of complication following discharge *via* face-to-face interview using a specially developed risk assessment tool. The questionnaire was designed to include as many factors as possible that may impact on postnatal health, as determined by existing literature, including the Saving Mothers Report (3). The questionnaire was divided into sections, covering sociodemographic information, pregnancy and pre-existing conditions, complications during labor and birth, pregnancy outcomes and mental health risk factors. To elicit risk factors for depression, anxiety or suicide risk, the Risk Factor Assessment developed by the Perinatal Mental Health Project (PMHP) in Cape Town was used (14). This screening tool was developed by the PMHP based on literature and their extensive experience with mental health disorders during pregnancy and soon after.

### File review

File reviews were conducted in all cases of pregnancy loss or neonatal death. This was done to determine timing of the death, classified as stillbirth, early neonatal or unclassified.

### Data management

Demographic variables included age, income, education, and foreign national status. Age was measured in years and categorized as below 18 years, 18 to 34 years, and 35 years and older. A binary variable for young and advanced maternal age were included in inferential analysis, young as less than 18 years, and advanced, 35 years or older. Income was analysed as a binary variable, no income or any income, for inferential analyses. Education was categorized as did not complete or completed grade 12/ secondary education. Health related factors included HIV status, hypertension during pregnancy (gestational hypertension or chronic hypertension before pregnancy), maternal pre-existing chronic conditions, and mental health risk factors (one or more risk factors, compared to no risk factors). These were included as binary variables. Pregnancy related factors included having four or more pregnancies, vaginal bleeding during pregnancy and no antenatal care (not booked). These were also included as binary variables in analysis. Birth related factors included birth out-of-facility, problems during labor, preterm labor, cesarean birth and perineal problems. All birth related factors were included as binary variables. All baby factors, which included low birthweight (<2,500 g), high birthweight (4,000 g or over) and resuscitation at birth were included as binary variables. Preterm birth was defined as birth before 37 completed weeks' gestation. Post term birth was defined as more than 41 weeks completed gestation. Apgar scores were defined as low, less than eight, or not low, eight and above.

### Analysis

We present descriptive statistics (proportion and frequency) for all risk factors measured using the risk assessment tool. For the inferential analysis, we focused on two key risk factors, HIV and hypertensive disease, the key outcome experienced by our participants, neonatal death, and high-risk mental health status. We examined associations between HIV and hypertensive disease (the key risk factors) and pregnancy outcomes, because these two pre-existing conditions are leading causes of maternal death in South Africa, including in the postnatal period (3). They are also identifiable and manageable in primary care settings. We examined these associations using Fishers Exact tests (due to commonly occurring expected frequencies below five). We did not identify any maternal deaths during our study but did identify stillbirths and neonatal deaths. We conducted logistic regression to assess associations between pregnancy loss and potential risk factors. We also conducted logistic regression to examine associations between the presence of mental health risk factors and other risk factors, because mental health is so often neglected in postnatal care. Factors that were associated with the dependent variable using a cut-off of *p* < 0.10 were included in multivariable analysis. The analysis was conducted using STATA 14 (15).

### Relevant guidelines

The South African Guidelines for Maternity Care (16) make provision for discharge from hospital 6 hours after birth provided certain conditions are met, and there are no medical concerns. For an uncomplicated caesarean birth, discharge is between 36- and 48-hours post-birth. All mothers should visit their local primary health clinic between 3 and 6 days after birth, to check on the health of both mother and baby. The next scheduled clinic visit is at 6 weeks, again for both mother and baby.

The South African Guidelines for the Prevention of Mother to Child Transmission of Communicable Infections (17) require an HIV viral load be taken at 3 months post-initiation for women newly initiated on ART, and at first antenatal visit for those already on ART. All women should have a viral load at birth, irrespective of when their last viral load test was done. A viral load result of over 1,000 copies per ml should trigger a repeat viral load followed by consideration of a second line ART regimen, as well as placing the infant on a high-risk ARV prophylaxis regimen. At discharge, mothers should be given two months' supply of ART. Breastfeeding is recommended for all mothers, regardless of HIV status. Infants of HIV positive mothers are tested for HIV at birth, and again at 10 weeks, 6 months, 18 months and after cessation of breastfeeding. The minimum duration of infant prophylaxis is 6 weeks in low-risk mothers, extended to 12 weeks or longer in high-risk situations.

### Ethical approvals

This study was approved by the Human Science Research Council Human Research Ethics Committee, reference REC 5/21/08/19, and the Limpopo Province Research Committee. Written consent was obtained from all participants.

## Results

### Descriptive statistics

#### Socio-demographic and antenatal profile

The risk questionnaire was completed for 882 mothers at the time of discharge. For these mothers, there were 867 live births. Sixteen mothers had twin births (1.8%), and there were 31 stillbirths (3.5%). One third of mothers reported living with their partner, 62% with family and 2% lived alone. Only 40% had completed secondary education, and only 12% reported formal employment (see Table 1). All the mothers planned to stay in the district for the six weeks following discharge. The median age of participants was 27 years, ranging from 14 to 48 years.

**Table 1:** Socio-demographic and antenatal profile of mothers receiving postnatal care at a rural district hospital in Limpopo Province, South Africa, August 2020–January 2021.

Characteristic	Number	Percentage	95% CI
Demographic characteristics				
Age	Under 18 years	21	2.4	1.6; 3.6
	18–34 years	691	78.3	75.5; 80.9
	35 years and older	170	19.3	16.8; 22.0
Education	Completed Grade 12/ secondary level	354	40.2	37.1; 43.5
	Did not complete Grade 12/ secondary level	527	59.8	56.5; 62.9
Income	Social grant	396	44.9	41.6; 48.2
	Employed	105	11.9	9.9; 14.2
	Family income	426	48.3	45.0; 51.6
	Informal work	11	1.2	0.7; 2.2
	No income	20	2.3	1.5; 3.5
Foreign national	Yes	41	5.6	3.4; 6.3
	No	841	95.4	93.7; 96.6
Pre-existing conditions				
Hypertension	Chronic hypertension	20	2.3	1.6; 3.6
	Gestational hypertension	46	5.2	3.9; 6.9
	Pre-eclampsia	2	0.2	0.1; 0.9
	Eclampsia	1	0.1	0.0; 0.8
	Any hypertensive disorder	69	7.9	6.3; 9.9
Chronic illnesses	Asthma	1	0.0	0.0; 0.1
	Diabetes	2	0.0	0.0; 0.1
	Epilepsy	3	0.0	0.0; 0.1
	Malaria	1	0.0	0.0; 0.1
	Mental health disorder	1	0.0	0.0; 0.1
	Substance abuse (alcohol)	1	0.0	0.0; 0.1
HIV status	HIV positive	201	22.8	20.1; 25.7
	HIV negative, tested at birth	28	3.2	2.2; 4.6
	HIV negative, not tested at birth	653	74.0	71.0; 76.8
Timing of HIV diagnosis	Before pregnancy	112	55.7	48.7; 62.5
	During pregnancy, before 36 weeks	82	40.8	34.2; 47.8
	During pregnancy, 36 weeks or after	7	3.5	1.7; 7.2
ART status	HIV positive, not on ART	4	2.0	0.7; 5.2
	HIV positive, on ART	197	98.8	94.8; 99.3
Most recent viral load timing	Before pregnancy	1	0.8	0.1; 5.9
	More than 6 months before birth	10	8.4	4.5; 15.0
	3–6 months before birth	26	21.9	15.2; 30.3
	Less than 3 months before birth	72	60.5	51.3; 69.0
	Date of birth	7	5.9	2.8; 11.9
	After birth	3	2.5	0.8; 7.6
Most recent viral load result	<50	64	62.1	52.3; 71.1
	50–399	24	23.3	16.0; 32.6
	400–999	1	1.9	0.5; 7.6
	≥1,000	13	12.6	7.4; 20.7
Antenatal				
Antenatal care	Unbooked – no antenatal visit	28	3.2	2.2; 4.6
	Booked – at least one antenatal visit	854	96.8	95.4; 97.8
Rapid plasma reagin (syphilis)	Positive	2	0.0	0.0; 0.1
	Negative	872	99.8	99.1; 99.9
Multiparous	4 or more pregnancies	185	21.0	18.4; 23.8
	Fewer than 4 pregnancies	695	79.0	76.2; 81.6
Antenatal Vaginal Bleeding	Yes	4	0.0	0.0; 0.1
Multiple pregnancy	Multiple	16	1.8	1.1; 2.9
MUAC^a^	Less than 23	38	4.3	3.1; 5.9
	Between 23 and 32	402	45.6	42.3; 48.9
	Greater than 32	110	12.5	10.4; 14.8
	Unknown	332	37.0	34.5; 40.8

^a^ MUAC -Mid-Upper Arm Circumference.

Antenatal Care coverage was high in our participants, with 854 women having received any antenatal care (96.8%). Twenty-eight (3.1%) women reported no antenatal care, placing them at high risk of poor outcomes. Eleven of the women who received no antenatal care were HIV positive (39.3%), and of them, seven (63.6%) had been diagnosed with HIV prior to the pregnancy. One hundred and eighty-five women (21.0%) reported being multiparous, with 4 or more pregnancies.

As shown in Table 1, 20 mothers (2.3%) had chronic hypertension (HPT), with an additional 49 mothers (5.6%) developing a hypertensive disorder during pregnancy. One hundred and twelve (12.7%) mothers had been diagnosed with HIV prior to the pregnancy, and an additional 89 (10.1%) tested positive during pregnancy (82 before 36 weeks gestation (9.3%), and 7 (0.8%) after). Overall, HIV prevalence was 22.8% [201 Women living with HIV (WLHIV)]. Viral load results were available for 103 women (51.2% of WLHIV). The most recent viral load result was greater than 1,000 copies/ml in 13 mothers (13% of those with an available result)- of these 11 had their most recent viral load drawn within 3 months of birth, and 2 were drawn between three and six months prior to birth. Twenty-one per cent of women with results available (*n* = 8) who had been diagnosed during pregnancy were virally unsuppressed (greater than 1,000 copies/ml), compared with 8% of women (*n* = 5) diagnosed before pregnancy. In 78 mothers (38.8% of WLHIV) there was no record of a viral load being done, and no result available. Only 4 women (2.0% of WLHIV) received more than one month supply of ART on discharge. Only two babies born to WLHIV did not have a birth polymerase chain reaction (PCR) recorded (1.0%), but of those with a PCR done only two (1.0%) had a result available before discharge. Most babies born to WLHIV received six weeks of nevirapine (182, 93.3%) although 13 received a single dose (6.7%). No mothers or babies were diagnosed with COVID-19 during their pregnancies or births.

#### Labour, birth and pregnancy outcomes

Two hundred and nineteen women (24.8%) had caesarean births, as seen in Table 2. There were 31 stillbirths and early neonatal deaths (3.5%), and 16 twin births, resulting in 866 live births. 263 babies (31.3%) were delivered before 37 completed weeks of pregnancy (see Table 2). We recorded 10 early neonatal deaths (11.5 per 1,000 live births). Three babies were born weighing less than 1500 g, with one of these less than 1000 g. In many cases the indication for caesarean births was unclear from the notes and interview.

**Table 2:** Labor, birth and pregnancy outcomes in mothers receiving postnatal care at a rural district hospital in Limpopo Province, South Africa, August 2020–January 2021.

Labor and birth	Number	Percentage	95% CI
Type of birth	Caesarean birth	219	24.8	22.1; 27.8
	Assisted birth	0	0.0	-
Prior to labor	Breech position	7	0.8	0.4; 1.7
	Rhesus negative	1	0.0	0.0; 0.1
During labor	Prolonged ROM	8	0.9	0.5; 1.8
	Foetal distress	13	1.5	0.9; 2.5
	Poor progress	6	0.7	0.3; 1.5
	Meconium-stained liquor	4	0.4	0.1; 1.2
	Prolonged second stage	8	0.9	0.5; 1.8
	Cephalopelvic disproportion	1	0.0	0.0; 0.1
	Ruptured placenta	1	0.0	0.0; 0.1
During & after birth	Perineal tear	179	20.3	17.8; 23.1
	Episiotomy	97	11.0	9.1; 13.2
	Hb < 10 after labour	18	2.0	1.3; 3.2
Baby				
Gestational age (completed weeks)	< 37 weeks	263	31.3	28.3; 34.5
	37–41 weeks	537	63.9	60.6; 67.1
	> 41 weeks	10	1.2	0.6; 2.2
	Unknown	30	3.6	2.5; 5.1
Weight	<1,000 g	2	0.0	0.0; 0.8
	1,000–1,499 g	2	0.0	0.0; 0.8
	1,500–2,499 g	115	13.4	11.2; 15.9
	2,500–3,999 g	714	83.4	80.7; 85.8
	4,000 g and above	23	2.7	1.7; 4.0
Low Apgar score		5	0.6	0.2; 1.4
Resuscitation needed		13	1.5	0.9; 2.6
Any death		41	4.6	3.4; 6.2
	Stillbirth	27	3.1	2.1; 4.4
	Early neonatal death	10	1.1	0.6; 2.1
	Death unclassified	4	0.5	0.1; 1,2

#### Mental health risk assessment

216 women (24.5%) had a mental health risk factor, with 40 reporting more than one (4.5%). As shown in Table 3 the most common risks were previous miscarriage in 112 women (12.7%), difficult relationship with their mother (57 women, 6.5%) and not being together with the baby's father (56 women, 6.3%).

**Table 3:** Mental health risk screen in mothers receiving postnatal care at a rural district hospital in Limpopo Province, South Africa, August 2020–January 2021.

Question	Number (*N* = 882)	%	95% CI
Feel pleased about having a new baby (answer: no)	2	0.2	0.0; 0.1
Had difficult things happen in the last year	12	1.4	0.1; 2.4
Not together with partner	56	6.3	4.9; 8.2
Partner doesn’t care about me	1	0.0	0.0; 0.1
Someone in household sometimes violent towards me	5	0.6	0.2; 1.4
Family and friends don’t care about how I feel	2	0.2	0.0; 0.9
Past experience of abuse	6	0.7	0.3; 1.5
Family and friends help in practical ways (answer: no)	3	0.3	0.1; 10.5
Don’t have a good relationship with mother	57	6.5	5.0; 8.3
Experienced miscarriage, stillbirth or death of a child	112	12.7	10.7; 15.1
Experience of serious depression, panic attacks or anxiety	9	1.0	0.5; 2.0

### Inferential analysis

#### HIV and hypertension

As shown in Table 4, deaths (*p* = 0.04) and low Apgar scores (*p* = 0.05) were more common for mothers with a hypertensive disorder, and babies with low birth weight (below 2500 g) were recorded more often in women with HIV (*p* = 0.04).

**Table 4:** Associations between pregnancy outcomes, HIV and HPT in mothers receiving postnatal care at a rural district hospital in Limpopo Province, South Africa, August 2020–January 2021.

Outcomes for baby	Number	%	Maternal HIV	%	*p*-value	Maternal HPT^a^	%	*p*-value
Any death	41	4.6	11	5.5	0.57	7	10.1	0.04
Stillbirth	27	3.1	9	4.5	0.24	4	5.8	0.26
Early neonatal death	10	1.1	2	1.0	0.99	2	2.9	0.19
Death unclassified	4	0.5						
Preterm birth (gestational age <37 completed weeks)	263	29.8	61	30.3	0.79	25	36.1	0.16
Gestational age >41 completed weeks	10	1.1	2	1.0	0.99	1	1.4	0.54
Twins	16	1.8	5	2.5	0.38	1	1.4	0.99
Birth weight <2,500 g	119	13.5	34	16.9	0.04	11	15.9	0.45
Birth weight >4,000 g	23	2.6	3	1.5	0.32	1	1.4	0.99
Low Apgar score	5	0.6	0	0.0	0.59	2	2.9	0.05
Resuscitation needed	13	1.5	2	1.0	0.74	2	2.9	0.25

^a^ HPT- hypertension.

#### Stillbirths and neonatal deaths

Table 5 shows associations between any death and potential explanatory factors. Unadjusted, stillbirth or early neonatal death was significantly associated with no antenatal care, high maternal age and any hypertensive disorder. In the adjusted multivariable analysis, only the association with no antenatal care remained significant (Odds ratio [OR] 3.6; 95% Confidence Interval [CI] 1.2; 11.0).

**Table 5:** Associations stillbirth or early neonatal death and explanatory factors in mothers receiving postnatal care at a rural district hospital in Limpopo Province, South Africa, August 2020–January 2021.

	Any death	Live baby	OR unadjusted	*p*-value	OR adjusted	*p*-value
No income	2	18	2.3 (0.5; 10.5)	0.26	−	
No Grade 12	23	504	0.9 (0.5; 1.6)	0.63	−	
Unbooked	4	24	3.7 (1.2; 11.2)	0.02	3.6 (1.2; 11.0)	0.03
HIV positive	11	190	1.3 (0.6; 2.6)	0.53	−	
High maternal age	13	157	2.0 (1.0; 4.0)	0.04	1.9 (0.9; 3.7)	0.08
Rapid plasma reagin	0	2	−		−	
Any HPT^a^	7	63	2.5 (1.1; 6.0)	0.03	2.2 (0.9; 5.4)	0.07

^a^ HPT- hypertension, Adjusted model includes unbooked, high maternal age and any hypertension.

#### Mental health

Table 6 shows associations between the presence of a mental health risk factor, and other identified risk factors. The presence of a mental health risk factor was significantly associated with having no income, not having matriculated, receiving no antenatal care, having HIV, advanced maternal age, and any hypertensive disorder, see Table 6. In multivariable analysis, having no income (OR 4.6, 95% CI 1.8; 11.7), no antenatal care (OR 2.4, 95% CI 1.1; 5.3), having HIV (OR 2.2, 95% CI 1.5; 3.1) and any hypertensive disorder (OR 1.7, 95% CI 1.0; 2.9) remained significant.

**Table 6:** Factors associated with the presence of mental health risk in mothers receiving postnatal care at a rural district hospital in Limpopo Province, South Africa, August 2020–January 2021.

	Mental health risk screen					
	One/more positive answers	No positive answers	OR crude	*p*-value	OR adjusted	*p*-value
No income	12	8	4.8 (2.1; 12.0)	0.01	4.6 (1.8; 11.7)	0.01
No grade 12	142	385	1.4 (1.0; 1.9)	0.04	1.2 (0.9; 1.7)	0.25
Unbooked	13	15	2.8 (1.3; 5.9)	0.01	2.4 (1.1; 5.3)	0.03
HIV positive	75	126	2.3 (1.6; 3.2)	<0.001	2.2 (1.5; 3.1)	<0.001
High maternal age	52	118	1.5 (1.0; 2.1)	0.04	1.2 (0.8; 1.8)	0.42
Rapid plasma reagin	2	0	−		−	
Any HPT^a^	24	46	1.7 (1.0; 2.8)	0.05	1.7 (1.0; 2.9)	0.06

^a^ HPT- hypertension. Adjusted model includes no income, no grade 12, unbooked, HIV positive, high maternal age and any hypertension.

## Discussion

Women and babies in this rural South African district experienced multiple risk factors for poor outcomes in the postpartum period. Important risk factors included low rates of secondary education and employment, high-risk comorbid conditions like HIV and hypertension, and high rates of preterm births. Although most of the women living with HIV were on ART, aspects of the PMTCT guidelines were not optimally implemented. These findings add to calls to strengthen postnatal care provision and point to important focus areas.

HIV remains the leading cause of maternal death in South Africa (3). In our sample, HIV prevalence was 23%, similar to the estimated prevalence for Limpopo province in the 2017 national Antenatal HIV Prevalence Survey (23.4%), and lower than the estimated national prevalence of 30.7% (18). ART coverage in our study was very high at 98%, but four mothers with HIV reported not being on ART. More than half of all women were diagnosed with HIV prior to the relevant pregnancy, providing an opportunity to achieve viral suppression prior to conception. Consistent with previous studies (19) viral suppression rates were poorer in women initiating ART during pregnancy, emphasising the importance of HIV testing services and ART programmes for women outside of antenatal care. It is also important that health care workers in primary care are able to advise women living with HIV wishing to become pregnant on how to do so safely (20).

Perinatally acquired HIV infection is also an important cause of neonatal and infant mortality. We found HIV testing at birth and adherence to viral load guidelines to be sub-optimal, which could impact on prevention of mother to child transmission. Coverage of early infant HIV testing was high although, as expected, very few results were available prior to discharge, creating the potential for HIV positive babies to be lost to care before ART is initiated. Point of care testing could potentially assist with this gap. Infant prophylaxis was appropriate in most cases, although 13% of exposed babies received single dose nevirapine, which is less effective than the recommended minimum of 6 weeks of nevirapine. PMTCT programmes have been more focused on antenatal services historically but need to shift to including the birth and postnatal periods to address the current transmission burden. In rural areas in particular, a high proportion of births occur at district hospitals, making them important implementers of the PMTCT programme. Improved adherence to national PMTCT guidelines, including HIV testing at birth, viral load testing, and active follow up and management of viral load results would have a positive impact on the health of mothers and babies in the postnatal period.

Hypertension also remains a leading cause of maternal death (3). At the time of discharge, 8% of women had been diagnosed with any hypertensive disorder, 2.3% with pre-existing chronic hypertension, and 49 mothers (5.6%) with a hypertensive disorder of pregnancy. Having hypertension was significantly associated with pregnancy loss or early neonatal death and low Apgar scores. In multivariable analysis, the odds of pregnancy loss were 2.2 times higher in women with hypertension. In addition, the odds of having a mental health risk factor were 1.7 times higher in women with hypertension. Chronic hypertension is seriously underdiagnosed and undertreated in South Africa, with estimates of unmet care need as high as 91% (21). Hypertension management needs to be strengthened across the life course for South African women, including in the postnatal period.

We found this population of women to be at high risk for postnatal mental health disorders due to socioeconomic and individual factors. Poverty is a known risk factor (7) and notably only 40% of women in our study had completed secondary level education and only 12% were employed. We also found that a quarter of women had a raised likelihood of depression, anxiety or suicide risk, using a mental health risk screening tool. In multivariable analysis this was associated with having no income, no antenatal care, HIV and any hypertensive disorder. When untreated, common mental disorders have serious consequences for mothers and babies in the postnatal period. Not only is quality of life impaired, but physical health and utilization of health services is affected, impacting on both mother and baby (7). Mental health screening and care should be included in all antenatal and postnatal care programmes, and integration into chronic disease services would be beneficial for this population.

It is unknown exactly how common preterm birth is in South Africa due to the multiple challenges in definition and measurement (22). Global estimates are approximately 10% of births, although there are major inequities between countries. We found that 30% of babies were assessed to have a gestational age below 37 completed weeks, the definition of premature birth, while 14% of babies were considered to be low birth weight, born weighing less than 2500 g. A cohort study in an urban South African district found rates of preterm birth and low birth weight of 16.4% and 13.5% (23). Our findings point to two concerns. Firstly, the reported rate of preterm birth is very high in this sample. Low socioeconomic status is a risk factor for preterm birth, so rates may be higher in rural areas. However, secondly, it may be that this rate is falsely inflated, due to challenges accurately assessing gestational age, especially when women present for antenatal care late in pregnancy. As premature birth is such an important risk factor for neonatal mortality (22), it is important that resources and additional support are provided to the babies who need it, requiring accurate assessment of gestational age.

This study was limited in that we collected data from health records available at discharge and supplemented this data with self-reports. Data from health records is frequently incomplete, and self-reported data may not be completely reliable. We suspect that we underreport labor and birth complications, possibly due to poor record keeping, which impacts on the ability of other levels of care to adequately manage patients. In addition, not all women who deliver do so at district hospitals. Complicated births are frequently referred to higher levels of care. However, these women are usually referred back and discharged from the district hospital, in which case they were included in our sample. Through programme data, we estimate that 80% of births happening in the sub-district occur at the district hospital. In addition, we were unable to include mothers under 18 years old in our study without parental consent. We had to exclude 15 young women whose parents and guardians were unavailable to give consent, resulting in the inclusion of 21 participants under 18 years old. Our programme data shows that 14.4% of pregnancies in Mopani District in 2021 were in young women aged 15–19 years, compared with 9.5% in our sample. If we had included the 21 young women, the proportion would have been 11.9%. Antenatal care was collected as a dichotomous variable, and we are unable to include the timing or number of visits in our reporting or analysis.

Postnatal care is known to be an important strategy to reduce maternal and neonatal deaths (3), and South African guidelines stipulate a 3–6-day visit for mothers at primary health care facilities. However, considering the ongoing high maternal and infant mortality in this period, as well as the risk factors experienced by discharged mothers and babies described in this paper, there is a need to improve postnatal care in this setting. Although guidelines pertaining to this period are largely aligned with international best practice, there is room for strengthening implementation. Research should focus on strengthening postnatal care in order to address the dominant risks to mothers and babies, including socioeconomic challenges, HIV and hypertension, and risks to mental health. Tools to identify mothers and babies at risk of postnatal complications would allow limited resources to be allocated where they are most needed.

## Data Availability

The raw data supporting the conclusions of this article will be made available by the authors, without undue reservation.
